# Supplementary material for: Kapitza-resistance-like exciton dynamics in atomically flat MoSe2-WSe2 lateral heterojunction
Source: Nat Commun. 2023 Sep 21;14:5881. doi: 10.1038/s41467-023-41538-6 (PMC10514293; doi:10.1038/s41467-023-41538-6)
Supplement: Supplementary file 1 — Supplementary Information [file 41467_2023_41538_MOESM1_ESM.pdf]

Kapitza-resistance-like exciton dynamics in atomically flat $MoSe_2$-WSe_2 lateral heterojunction

Hassan Lamsaadi[1,†] Dorian Beret[2,†] Ioannis Paradisanos[2,9,†] Pierre Renucci[2,†] Delphine Lagarde[2,†] Xavier Marie[2,†] Bernhard Urbaszek[2,5,†] Ziyang Gan [3,†] Antony George[3,4,†] Kenji Watanabe[7,†] Takashi Taniguchi[8,†] Andrey Turchanin[3,4,†] Laurent Lombez[2,*,†] Nicolas Combe[1,†] Vincent Paillard[1,†] and Jean-Marie Poumirol[1*,†]

†[1] *CEMES-CNRS, Université de Toulouse, Toulouse, France*

‡[2] *Université de Toulouse, INSA-CNRS-UPS, LPCNO, 135 Avenue Rangueil, 31077 Toulouse, France*

¶[3] *Friedrich Schiller University Jena, Institute of Physical Chemistry, 07743 Jena, Germany*

§[4] *Abbe Centre of Photonics, 07745 Jena, Germany*

‖[5] *Ulm University, Central Facility of Electron Microscopy, D-89081 Ulm, Germany*

⊥[5] *Institute of Condensed Matter Physics, Technische Universität Darmstadt, Darmstadt, Germany*

#[7] *Research Center for Functional Materials, National Institute for Materials Science, 1-1 Namiki, Tsukuba 305-0044, Japan*

@[8] *International Center for Materials Nanoarchitectonics, National Institute for Materials Science, 1-1 Namiki, Tsukuba 305-0044, Japan*

△[9] *Institute of Electronic Structure and Laser, Foundation for Research and Technology-Hellas, Heraklion, 70013, Greece*

E-mail: laurent.lombez@insa-toulouse.fr; jean-marie.poumirol@cemes.fr

I - Modified exciton transfer near-field model

Our near-field model is based on the following assumptions :

(1) Bright excitons diffuse as classical quasi-particles.

(2) In the low density regime, we conserved that bright excitons are non-interacting with other type of excitons mainly: dark excitons, B-excitons, momentum forbidden excitons.

(3) The effective diffusion lengths L_1 and L_2 are quasi constant in both materials which are separated by a depletion zone acting as an ideal thin interface of fixed width $\epsilon \ll L_1$,L_2, where no electron-hole pair are created and no electron-hole recombination occurs.

(4) Presence of a local uniform field force $\vec{F}$ inside the interface ($-\frac{\epsilon}{2} \leq x \leq \frac{\epsilon}{2}$) due to the difference in the band-gaps of the two materials, leading to a drift velocity $\vec{v}^* = \mu_b \vec{F}$ which modifies locally the exciton velocity.

Determination of $\mathbf{n(x,x_{tip})}$

In the linear regime, we solve, analytically and separately, the 1D linear diffusion equation in each material i ($1 = WSe_2, 2 = MoSe_2$). The terms in the steady-state diffusion equation (1) given in the main text are homogenized to an excitonic density as follows:

$$L_i^2 \frac{d^2 n(x, x_{tip})}{dx^2} - n(x, x_{tip}) + n_{s_i} e^{-\frac{(x-x_{tip})^2}{w^2}} = 0 \tag{1}$$

Where $n_{s_i} = \Gamma_{0_i} \tau_i$. The solution of (1) is written as :

$$n(x > 0, x_{tip}) = C_1 e^{x/L_1} + C_2 e^{-x/L_1} - n_{s_1}/L_1 \int_x^{\infty} ds \sinh\left((s-x)/L_1\right) e^{-\frac{(s-x_{tip})^2}{w^2}} \tag{2}$$

$$n(x < 0, x_{tip}) = C_3 e^{x/L_2} + C_4 e^{-x/L_2} + n_{s_2}/L_2 \int_{-\infty}^{x} ds \sinh\left((s-x)/L_2\right) e^{-\frac{(s-x_{tip})^2}{w^2}} \tag{3}$$

Where C_1, C_2, C_3 and C_4 are integration constants. To determine them, first we impose boundary conditions as $n(x \to \pm\infty, x_{tip}) = 0$, resulting in $C_1 = C_4 = 0$. Second, we express C_2 and C_3 as a function of $n_0^{\pm} = n(x = 0^{\pm}) \simeq n(x = \pm\frac{\epsilon}{2})$ to obtain the following solutions:

$$n(x>0,x_{tip}) = \left(n_0^+ + n_{s_1}/L_1\int_0^{\infty} ds\sinh\left(s/L_1\right)e^{-\frac{(s-x_{tip})^2}{w^2}}\right)e^{-x/L_1} - n_{s_1}/L_1\int_x^{\infty} ds\sinh\left((s-x)/L_1\right)e^{-\frac{(s-x_{tip})^2}{w^2}} \quad (4)$$

$$n(x<0,x_{tip}) = \left(n_0^- - n_{s_2}/L_2\int_{-\infty}^{0} ds\sinh\left(s/L_2\right)e^{-\frac{(s-x_{tip})^2}{w^2}}\right)e^{x/L_2} + n_{s_2}/L_2\int_{-\infty}^{x} ds\sinh\left((s-x)/L_2\right)e^{-\frac{(s-x_{tip})^2}{w^2}} \quad (5)$$

In order to obtain the steady-state expressions of $n_0^{\pm}$ we first calculate, using (4 - 5), the exciton flux at each side of the interface as follows :

$$j_n(x=0^+) = -D_1\left.\frac{dn}{dx}\right|_{x=0^+} = \frac{L_1}{\tau_1}n_0^+ - \frac{n_{s_1}}{\tau_1}\int_0^{\infty} dx e^{-x/L_1}e^{-\frac{(x-x_{tip})^2}{w^2}} \quad (6)$$

$$j_n(x=0^-) = -D_2\left.\frac{dn}{dx}\right|_{x=0^-} = -\frac{L_2}{\tau_2}n_0^- + \frac{n_{s_2}}{\tau_2}\int_{-\infty}^{0} dx e^{x/L_2}e^{-\frac{(x-x_{tip})^2}{w^2}} \quad (7)$$

Second, we solve analytically, in the steady state, the semi-classical diffusion equation of bright exciton inside the interface given by :

$$\vec{\nabla}.\vec{j}_n = \vec{\nabla}.(n\vec{v}^* - D_b\vec{\nabla}n) \simeq 0 \quad (8)$$

Where $n\vec{v}^*$ is the local drift exciton flux and D_b is the diffusion coefficient inside the interface. By imposing the continuity of the exciton density $n(-\frac{\epsilon}{2} \leq x \leq \frac{\epsilon}{2})$ inside the interface, we demonstrate that the junction imposes a uniform local density of exciton flux $\vec{j}_n = n\vec{v}^* - D_b\vec{\nabla}n$ on both sides of the interface, which depends on the tip position. Integrating (8) over the interface width allows a link between j_n and $n_0^{\pm}$ as follows :

$$n_0^+ = \left(n_0^- + \frac{j_n}{v^*}\right)e^{-\frac{v^*\epsilon}{D_b}} - \frac{j_n}{v^*} \quad (9)$$

By replacing in (9) the exciton flux $j_n = j_n(x=0^{\pm})$ obtained in (6 - 7) we get the two independent relations between $n_0^{\pm}$ as follows :

$$n_0^+ = n_0^-\kappa^+ + \frac{n_{s_2}}{v^*\tau_2}\left(e^{-\frac{v^*\epsilon}{D_b}} - 1\right)\int_{-\infty}^{0} dx e^{x/L_2}e^{-\frac{(x-x_{tip})^2}{w^2}} \quad (10)$$

$$n_0^- = n_0^+/\kappa^- - \frac{n_{s_1}}{v^*\tau_1}\left(e^{\frac{v^*\epsilon}{D_b}} - 1\right)\int_0^{\infty} dx e^{-x/L_1} e^{-\frac{(x-x_{tip})^2}{w^2}} \tag{11}$$

Where $\kappa^{\pm}$ are expressed as follows :

$$\begin{cases} \kappa^+ = \left(1 - \frac{L_2}{v^*\tau_2}\right) e^{-\frac{v^*\epsilon}{D_b}} + \frac{L_2}{v^*\tau_2} \\ \kappa^- = \left[(1 + \frac{L_1}{v^*\tau_1})e^{\frac{v^*\epsilon}{D_b}} - \frac{L_1}{v^*\tau_1}\right]^{-1} \end{cases}$$

When the near-field excitation ($w \ll L_1$) takes place inside WSe_2, the integral in (10) becomes null, and $\kappa^+ = n_0^+/n_0^-$ describes the exciton diffusion from WSe_2 to $MoSe_2$. However, if the near-field excitation ($w \ll L_2$) takes places inside $MoSe_2$, the integral in (11) becomes null, and $\kappa^- = n_0^+/n_0^-$ describes the exciton diffusion from $MoSe_2$ to WSe_2. In both cases, the ideal junction maintains a local equilibrium of exciton densities on both sides of the interface, described by the constant $\kappa = n_0^+/n_0^-$, we call this constant the partition coefficient and define it as follows :

$$\kappa = \begin{cases} \kappa^+ = \left(1 - \frac{L_2}{v^*\tau_2}\right) e^{-\frac{v^*\epsilon}{D_b}} + \frac{L_2}{v^*\tau_2} & x_{tip} > 0 \\ \kappa^- = \left[(1 + \frac{L_1}{v^*\tau_1})e^{\frac{v^*\epsilon}{D_b}} - \frac{L_1}{v^*\tau_1}\right]^{-1} & x_{tip} < 0 \end{cases} \tag{12}$$

We can now, according to (10 - 11), obtain the complete expressions of $n_0^{\pm}$ as follows :

$$n_0^-(x_{tip}) = \frac{n_{s_1}}{\frac{\tau_1}{\tau_2}L_2 + \kappa^+ L_1}\int_0^{\infty} dx e^{-x/L_1} e^{-\frac{(x-x_{tip})^2}{w^2}} + \frac{n_{s_2}}{L_2 + \kappa^- \frac{\tau_2}{\tau_1}L_1}\int_{-\infty}^{0} dx e^{x/L_2} e^{-\frac{(x-x_{tip})^2}{w^2}} \tag{13}$$

$$n_0^+(x_{tip}) = \frac{n_{s_1}\kappa^+}{\frac{\tau_1}{\tau_2}L_2 + \kappa^+ L_1}\int_0^{\infty} dx e^{-x/L_1} e^{-\frac{(x-x_{tip})^2}{w^2}} + \frac{n_{s_2}\kappa^-}{L_2 + \kappa^- \frac{\tau_2}{\tau_1}L_1}\int_{-\infty}^{0} dx e^{x/L_2} e^{-\frac{(x-x_{tip})^2}{w^2}} \tag{14}$$

Integrated PL intensity

The PL intensities away from the junction are given by :

$$I^{\infty}_{PL}(A^{WSe_2}_{1s})(x_{tip} \to +\infty) = \int_0^{\infty} dx \frac{\Gamma_1(x, x_{tip})\tau_1}{\tau_1^r} = \sqrt{\pi}\frac{n_{s_1} w}{\tau_1^r} \tag{15}$$

$$I^{\infty}_{PL}(A^{MoSe_2}_{1s})(x_{tip} \to -\infty) = \int_{-\infty}^{0} dx \frac{\Gamma_2(x, x_{tip})\tau_2}{\tau_2^r} = \sqrt{\pi}\frac{n_{s_2} w}{\tau_2^r} \tag{16}$$

Experimentally we know that $\beta = I^{\infty}_{PL}(A^{WSe_2}_{1s})/I^{\infty}_{PL}(A^{MoSe_2}_{1s}) \simeq 3$, giving us $\frac{n_{s_1}\tau_2^r}{n_{s_2}\tau_1^r} \simeq 3$. We then use (4 - 5) to calculate the PL intensities as a function of the tip position as follows:

$$I_{PL}(A^{MoSe_2}_{1s})(x_{tip}) = \int_{-\infty}^{0} dx \frac{n(x, x_{tip})}{\tau_2^r} = \frac{n_0^-(x_{tip})}{\tau_2^r} L_2 + \frac{n_{s_2}}{\tau_2^r}\int_{-\infty}^{0} dx (1 - e^{x/L_2}) e^{-\frac{(x - x_{tip})^2}{w^2}} \tag{17}$$

$$I_{PL}(A^{WSe_2}_{1s})(x_{tip}) = \int_0^{\infty} dx \frac{n(x, x_{tip})}{\tau_1^r} = \frac{n_0^+(x_{tip})}{\tau_1^r} L_1 + \frac{n_{s_1}}{\tau_1^r}\int_0^{\infty} dx (1 - e^{-x/L_1}) e^{-\frac{(x - x_{tip})^2}{w^2}} \tag{18}$$

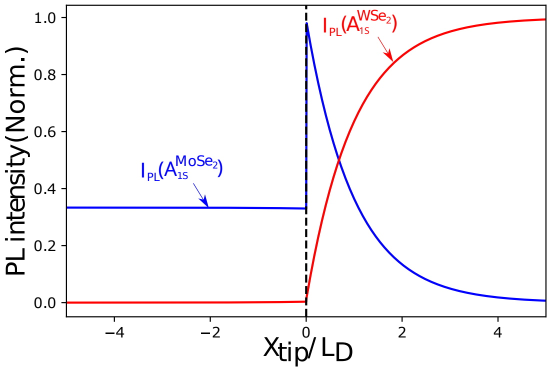

Figure 1: Normalized PL intensities calculated using as a simple example: $\beta = 3, \kappa = 0.001$, $L_1 = L_2 = L_D = 0.1\mu m$, $\tau_1 = \tau_2$ and $\tau_1^r = \tau_2^r$.

Fig. 1 shows the calculated PL intensities from (17 - 18). One can see that the calculated PL intensity of $MoSe_2$ is discontinuous at the junction.

Continuity of the integrated PL intensity

We observed experimentally that for both e-LH and un-LH systems, the integrated PL intensities of each material remain continuous as the tip crosses the junction. The continuity is not described by the model presented above that does not account for potential variations of the scattering time with positions. However, we know that the velocity of excitons interacting with the junction is modified (due to the drift velocity $\vec{v}^*$). For example, as the tip approaches the junction, excitons diffusing from $MoSe_2$ towards WSe_2 are stopped at the junction, their average velocity going to zero. We believe that this phenomenon, by reducing the probability to encounter defects and decreasing the kinetic energy carried by the excitons, has a local effect on the exciton non-radiative lifetime. As the total amount of excitons in the steady state depends on the scattering time, the excitonic density at the junction is increased, resulting in an enhanced PL intensity. This interaction does not affect the radiative life-time.

To account for this local variation of scattering time of the exciton interacting with the junction, for each tip position, we accounted for the resulting increase of the exciton density at the junction while the near-field excitation occurs inside $MoSe_2$ and rewrite (13 - 14), as follows:

$$n_0^-(x_{tip}) = \frac{n_{s_1}}{\frac{\tau_1}{\tau_2}L_2 + \kappa^+ L_1} \int_0^\infty dx e^{-x/L_1} e^{-\frac{(x-x_{tip})^2}{w^2}} + \frac{n_{s_2}\gamma}{L_2 + \kappa^- \frac{\tau_2}{\tau_1}L_1} \int_{-\infty}^0 dx e^{x/L_2} e^{-\frac{(x-x_{tip})^2}{w^2}} \tag{19}$$

$$n_0^+(x_{tip}) = \frac{n_{s_1}\kappa^+}{\frac{\tau_1}{\tau_2}L_2 + \kappa^+ L_1} \int_0^\infty dx e^{-x/L_1} e^{-\frac{(x-x_{tip})^2}{w^2}} + \frac{n_{s_2}\kappa^-\gamma}{L_2 + \kappa^- \frac{\tau_2}{\tau_1}L_1} \int_{-\infty}^0 dx e^{x/L_2} e^{-\frac{(x-x_{tip})^2}{w^2}} \tag{20}$$

The factor γ is determined by imposing the continuity of the PL intensity at the junction:

$$I_{PL}(A_{1s}^{MoSe_2})(x_{tip} = 0^+) = I_{PL}(A_{1s}^{MoSe_2})(x_{tip} = 0^-) \tag{21}$$

and

$$I_{PL}(A_{1s}^{WSe_2})(x_{tip}=0^+) = I_{PL}(A_{1s}^{WSe_2})(x_{tip}=0^-) \tag{22}$$

resulting in $\gamma = \frac{\frac{\tau_1}{\tau_2}L_2+\kappa^+L_1}{\frac{\tau_1}{\tau_2}L_2+\kappa^-L_1}\frac{\tau_2\tau_1^r}{\tau_1\tau_2^r}\beta$. In our case, $\kappa^+ \sim \kappa^- \ll 1$, thus $\gamma \simeq \frac{\tau_2\tau_1^r}{\tau_1\tau_2^r}\beta$. The normalized PL intensities can then be written as follows:

$$\frac{I_{PL}(A_{1s}^{MoSe_2})(x_{tip})}{I_{PL}^{\infty}(A_{1s}^{WSe_2})} = \frac{A_\kappa}{\sqrt{\pi}w}\int_0^\infty dx e^{-x/L_1}e^{-\frac{(x-x_{tip})^2}{w^2}} + \frac{1}{\sqrt{\pi}\beta w}\int_{-\infty}^0 dx\left(1+(\beta A_\kappa-1)e^{x/L_2}\right)e^{-\frac{(x-x_{tip})^2}{w^2}} \tag{23}$$

$$\frac{I_{PL}(A_{1s}^{WSe_2})(x_{tip})}{I_{PL}^{\infty}(A_{1s}^{WSe_2})} = \frac{1}{\sqrt{\pi}w}\int_0^\infty dx\left(1-(1-B_\kappa)e^{-x/L_1}\right)e^{-\frac{(x-x_{tip})^2}{w^2}} + \frac{B_\kappa}{\sqrt{\pi}w}\int_{-\infty}^0 dx e^{x/L_2}e^{-\frac{(x-x_{tip})^2}{w^2}} \tag{24}$$

Where $A_\kappa = \frac{L_2\tau_2}{L_2\tau_1+L_1\tau_2\kappa^+}\frac{\tau_1^r}{\tau_2^r} \simeq \frac{\tau_2\tau_1^r}{\tau_1\tau_2^r}$ and $B_\kappa = \kappa^+ A_\kappa \frac{\tau_2^r}{\tau_1^r}$. The radiative lifetimes are of the same order of magnitude in each material, we then get an order of magnitude of the partition coefficient $\kappa^+ \sim \kappa^- \sim B_\kappa/A_\kappa$.

Table 1: Results of the PL fits using the near-field model for both e-LH and un-LH systems.

**Area**	$L_1(nm)$	$L_2(nm)$	A_κ	B_κ	$\kappa^\pm \sim B_\kappa/A_\kappa$
e-LH	120 ± 6	110 ± 5.5	1.1	10^{-3}	$\sim 10^{-3}$
un-LH	550 ± 55	45 ± 45	1	10^{-3}	$\sim 10^{-3}$

Kapitza resistance equivalence

The abrupt change in the exciton energy at the junction causes a discontinuity of excitonic density. This finding can be interpreted in terms of an excitonic Kapitza resistance R_n whose sign depends on the direction of excitonic flux. Indeed, $R_n > 0$ for the diffusion from $MoSe_2$ to WSe_2 and $R_n < 0$ from WSe_2 to $MoSe_2$, illustrating the non-reciprocal excitonic diffusion through the junction. According to (9), we can define a Kapitza exciton resistance of a high-quality junction as a function of the partition coefficient κ as follows:

$$R_n = \frac{n(x=0^-) - n(x=0^+)}{j_n} \simeq \begin{cases} R_n^+ \simeq \frac{1-\kappa^+}{v^*} \frac{1-e^{v^*\epsilon/D_b}}{1-\kappa^+ e^{v^*\epsilon/D_b}} < 0 & x_{tip} > 0 \\ R_n^- \simeq \frac{1-\kappa^-}{v^*} \frac{1-e^{v^*\epsilon/D_b}}{\kappa^- e^{v^*\epsilon/D_b}-1} > 0 & x_{tip} < 0 \end{cases} \quad (25)$$

One can see that if the gap difference between the two TMD-MLs forming the junction is high ($v^* = \mu F \to \infty$), there is a strong influence on the exciton diffusion ($R_n^{\pm} \to \mp\infty$). On the other hand, in the opposite situation (very weak gap difference, compared to $k_B T$) $v^* \to 0$, the exciton density becomes continuous, and the junction loses its Kapitza resistance ($R_n \to 0$).

II - Power dependence transport properties of WSe_2

Here we describe the results obtained from the time-resolved PL profile in WSe_2 where we see the large change between the e-LH and un-LH samples. Spatio-temporal information allows us to separate the transport and the recombination mechanisms. The experiment is based on a diffraction-limited laser excitation that induces lateral diffusion of the photogenerated excitonic species. We used a Streak camera system to record the time evolution of the PL spatial profile $I_{PL} = I(x,t)$ with a time resolution of $5.5ps$. The Ti:Sa laser excitation is set to $E_{ex} = 1.79eV$, with a $80MHz$ repetition frequency, $1.5ps$ pulse width and we vary the excitation power from $10\mu W$ to $1mW$. Each PL spatial profile is considered as Gaussian, and we examine the squared width w^2 of the PL profile as a function of time. In case of (quasi)linear broadening, an effective diffusion coefficient D_{eff} can be extracted according to the relation $\Delta w^2(t) = 4D_{eff}t$.

We first present the results from uncapped sample(un-LH). Fig. 2 **A** shows TRPL data and the extracted lifetimes (Fig. 2 **B**) obtained from bi-exponential fit. The results show a reduction of the overall lifetime with increasing excitation power. Fig. 2 **C** shows the time evolution of the squared width w^2 where we see an increase of the slope as we increase the excitation power. We then extract the effective diffusion coefficient D_{eff} (Fig. 2 **C**) which is found to increase with the excitation power from a typical value in TMD of $1cm^2/s$ up to $20cm^2/s$.

Similar observations are found from the fully encapsulated sample e-LH as it can be

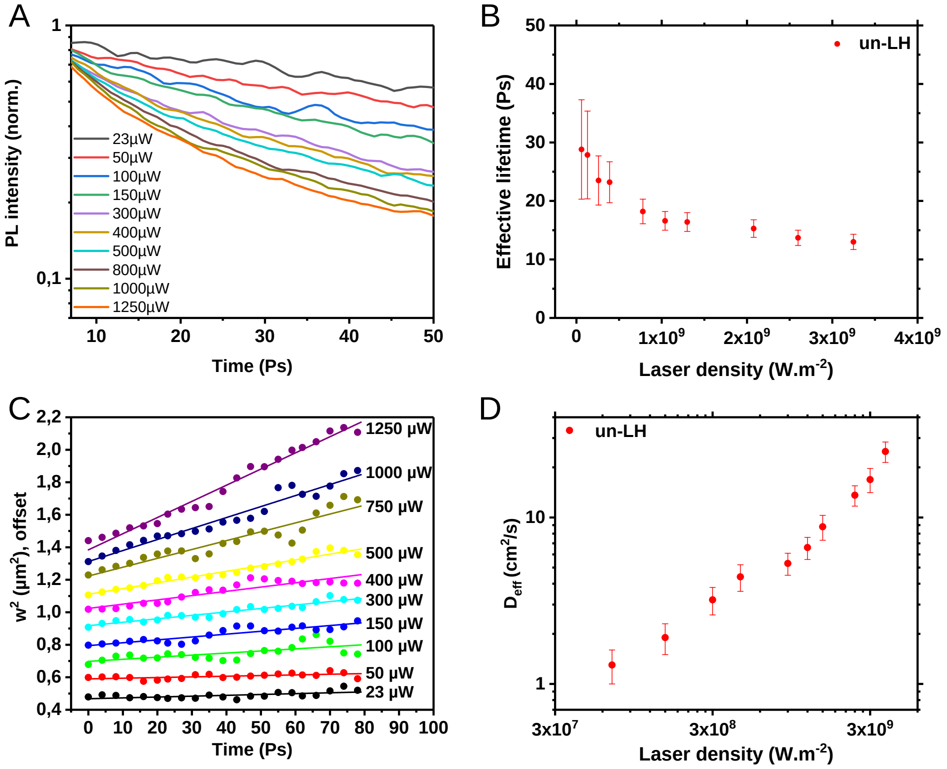

Figure 2: Experimental results of time resolved PL profile for the *un-LH sample*. (A) Time resolved PL intensity (TRPL) for various excitation powers. (B) Extracted lifetimes from TRPL data. (C) Time evolution of the squared width w^2, the curves for the different excitation powers are offset for visibility. (D) Extracted effective diffusion coefficient from (C) as a function of excitation power.

seen in Fig. 3. The increase of the effective diffusion coefficient with exciton density has been previously observed in the literature.[1,2] As mentioned in the main text, although differences between fully encapsulated sample and uncapped one were found in Ref.[2] , we observe a similar trend in the power dependence behavior in our two samples. This is probably due to the bottom hBN layer which prevents a large discrepancy of the transport mechanisms on the two samples.

To go further, we model the experimental results by solving numerically a classical diffusion equation:

$$D\frac{d^2n(x)}{dx^2} - \frac{n(x)}{\tau} - C_{EEA}n^2(x) + \Gamma(x,t=0) = \frac{dn(x)}{dt} \tag{26}$$

where $\Gamma(x, t = 0)$ is the local excitation generation rate at $t = 0ps$, $\tau = 100ps$ the effective

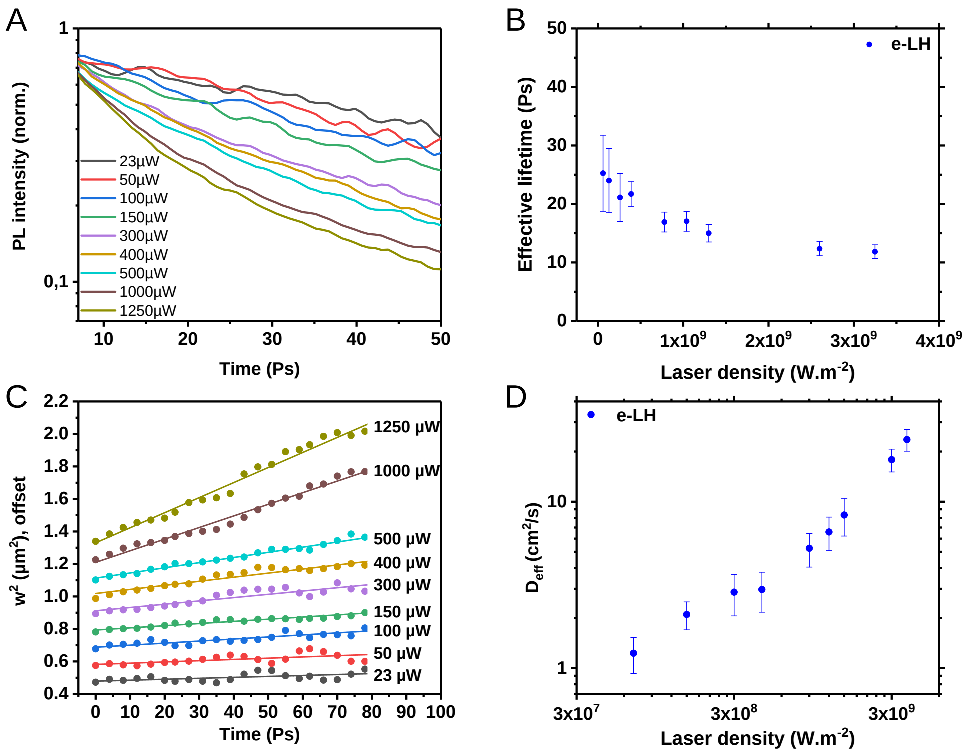

Figure 3: Experimental results of time resolved PL profile for the *e-LH sample*. (A) Time resolved PL intensity (TRPL) for various excitation powers. (B) Extracted lifetimes from TRPL data. (C) Time evolution of the squared width w^2, the curves for the different excitation powers are offset for visibility. (D) Extracted effective diffusion coefficient from (C) as a function of the excitation power.

lifetime which accounts for radiative and non-radiative decays, $D = 1cm^2/s$ is the intrinsic diffusion coefficient and C_{EEA} is the excitonic Auger coefficient (i.e. exciton-exciton annihilation EEA). We then calculated $I_{PL}(x,t) \propto n(x,t)$ from which we extract TRPL data and calculate $\Delta w^2(t) = 4D_{eff}t$. Results are displayed in Fig. 4 for a large set of $\Gamma(x, t = 0)$. We found a good agreement with the experimental data when we set $C_{EEA} = 0.2 \sim 10^{-6}cm^2/s$. Although we do not pretend to extract an exact Auger coefficient, its low value might confirm that the bottom hBN layer prevents the TMD layer to be influenced by dielectric disorder. Despite its small value, the EEA process influences the transport at high excitation flux and increases the effective diffusion coefficient D_{eff} as observed in the literature.[1,2]

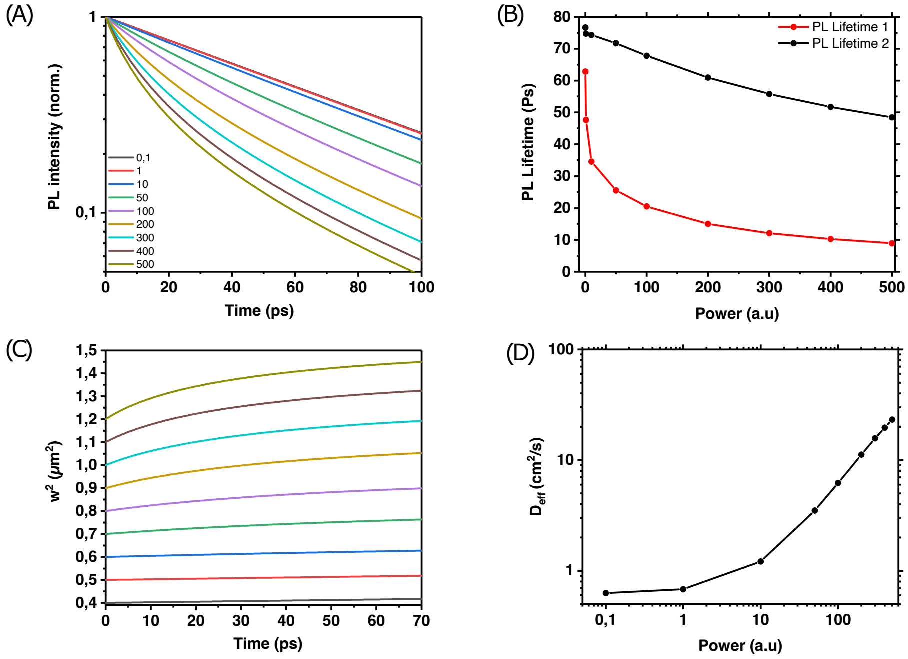

Figure 4: Modeling results of time resolved PL profile for the *e-LH sample.* (A) Time resolved PL intensity (TRPL) for various excitation powers. (B) Extracted lifetimes from TRPL data. (C) Time evolution of the squared width w^2, the curves for the different excitation powers are offset for visibility. (D) Extracted effective diffusion coefficient from (C) as a function of the excitation power.

III - Additional figures

To select ideal interfaces away from any defects (Inclusions, cracks, ...), we have also performed Raman spectroscopy measurements. Raman spectra are recorded every 500 nm using 532 nm wavelength and 1 mW power excitation of linearly polarized laser in both samples e-LH and un-LH. Fig. 5 **A** displays the typical µ-Raman spectra measured in e-LH (left) and un-LH (right). Each material has been identified by its pronounced Raman peak, either ${A_1'}^{MoSe_2}$ mode located at $\sim 241cm^{-1}$ and A_1'/E'^{WSe_2} mode located at $\sim 251cm^{-1}$. Some inclusions are visible in the PL maps shown in Fig. 1 of the main text but are not visible in the Raman maps shown here because their small size reduces significantly the Raman intensity.

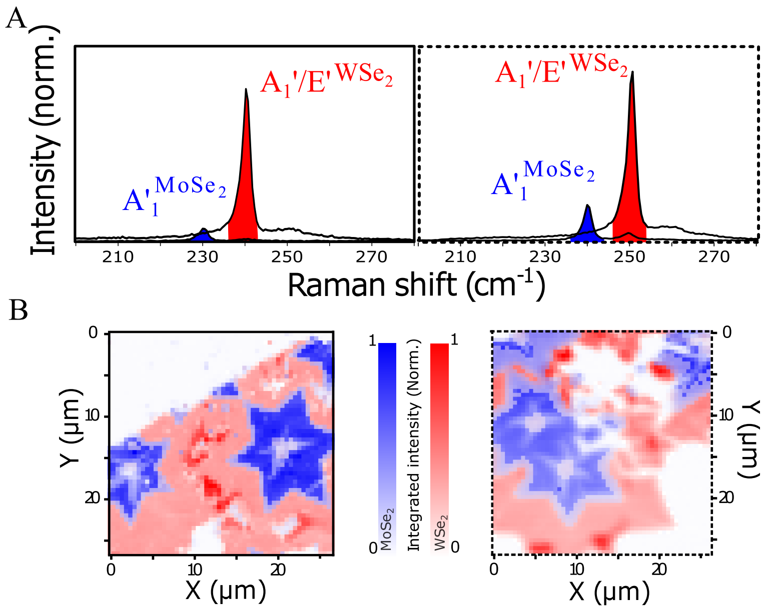

Figure 5: **A**. Typical µ-Raman spectra measured in the WSe_2 and $MoSe_2$ regions of e-LH (left panel) and un-LH (right panel). **B**. Spectrally integrated µ-Raman intensity maps of e-LH (left panel) and un-LH (right panel). The integrated intensity is obtained by integrating $MoSe_2$ (respectively WSe_2) µ-Raman spectra over the shaded blue (respectively red) range shown in A. µ-Raman spectra are recorded every 500 nm (step size), using a 532 nm excitation wavelength and 1 mW laser power.

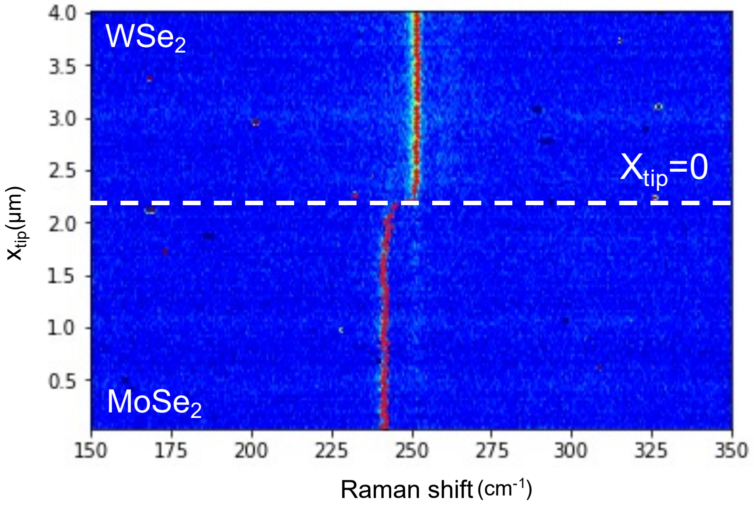

Figure 6: Tip enhanced Raman spectra intensity map versus position of the tip. The maximum of the pronounced Raman peak is marked by a red dot on the color map. The position of the interface is determined by pinpointing the x_{tip} position where the signal abruptly change from $MoSe_2$ Raman signature to WSe_2 Raman signature.

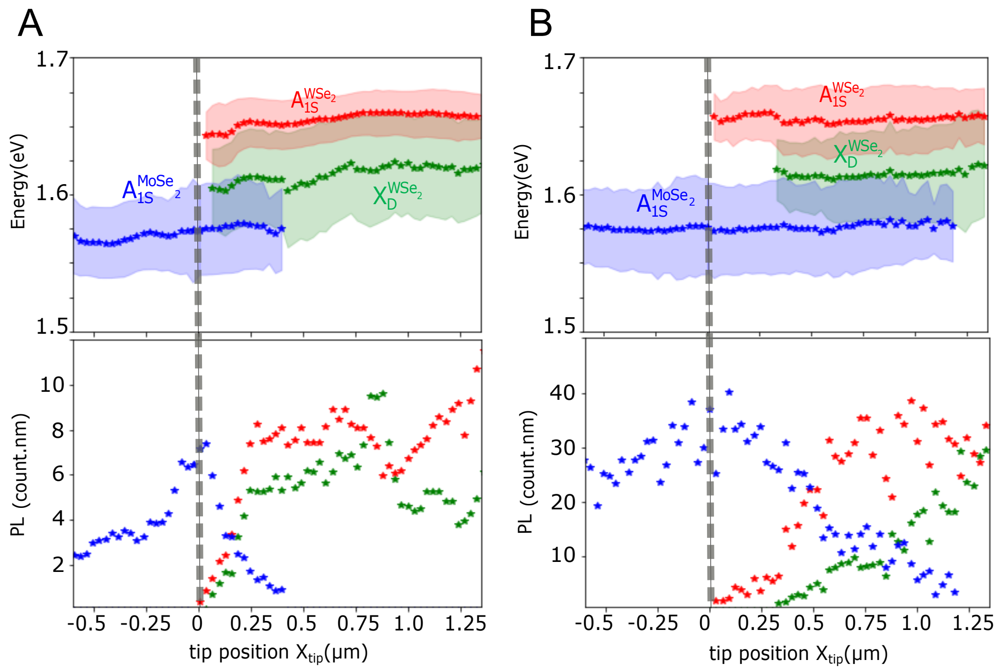

Figure 7: **A** (**B**) Top: Energy and FWHM of each Lorentzian peak obtained from the fitting procedure described in the main text. Bottom: Amplitude of each Lorentzian peak obtained from the fitting procedure. The red, blue and green stars indicate $A^{1s}_{WSe_2}$, $A^{1s}_{MoSe_2}$, and the WSe_2 dark exciton ($X^{D}_{WSe_2}$) respectively.

References

(1) Uddin, S. Z.; Higashitarumizu, N.; Kim, H.; Yi, J.; Zhang, X.; Chrzan, D.; Javey, A. Enhanced Neutral Exciton Diffusion in Monolayer WS $_2$ by Exciton–Exciton Annihilation. *ACS Nano* **2022**, *16*, 8005–8011.

(2) Zipfel, J.; Kulig, M.; Perea-Causín, R.; Brem, S.; Ziegler, J. D.; Rosati, R.; Taniguchi, T.; Watanabe, K.; Glazov, M. M.; Malic, E.; Chernikov, A. Exciton diffusion in monolayer semiconductors with suppressed disorder. *Physical Review B* **2020**, *101*, 115430.
